# Hybrid ^99m^Tc–ICG Sentinel Lymph Node Mapping in Apparent Early-Stage Epithelial Ovarian Cancer: A First Prospective Evaluation of a True Molecular Hybrid Tracer (HibrOv Trial)

**DOI:** 10.3390/cancers18121973

**Published:** 2026-06-17

**Authors:** Joana Amengual Vila, Catalina Maria Sampol Bas, Adriana Quintero Duarte, Ane Ugarteburu Pérez, Mario Ruiz Coll, Jorge Rioja Merlo, Anna Torrent Colomer

**Affiliations:** 1 Gynecologic Oncology Unit, Obstetrics and Gynecology Department, Hospital Universitari Son Espases, 07120 Palma, Spain; ane.ugarteburu@ssib.es (A.U.P.); mario.ruiz@ssib.es (M.R.C.); jorgeo.rioja@ssib.es (J.R.M.); atorrent@ssib.es (A.T.C.); 2 School of Medicine, Universitat de les Illes Balears (UIB), 07120 Palma, Spain; 3 Institut d’Investigació Sanitària de les Illes Balears, IdISBa, 07120 Palma, Spain; 4 Department of Nuclear Medicine, Hospital Universitari Son Espases, 07120 Palma, Spain; catalinam.sampol@ssib.es; 5 Department of Pathology, Hospital Universitari Son Espases, 07120 Palma, Spain; amquintero@ssib.es

**Keywords:** sentinel lymph nodes, epithelial ovarian cancer, early-stage ovarian cancer, hybrid tracer, technetium-99m nanocolloid, indocyanine green, surgical staging, lymphatic mapping, pelvic lymph nodes, para-aortic lymph nodes

## Abstract

**Background/Objectives**: Systematic lymphadenectomy is recommended in apparent early-stage epithelial ovarian cancer (EOC) to assess nodal status, but it is associated with significant morbidity and lacks survival benefit. Sentinel lymph node (SLN) mapping may offer a less invasive alternative, although evidence remains limited due to the complexity of ovarian lymphatic drainage and methodological heterogeneity across studies. This prospective study evaluates the feasibility and diagnostic accuracy of a true hybrid ^99m^Tc–indocyanine green (ICG) tracer for SLN mapping in apparent early-stage EOC. **Methods**: A prospective observational study was conducted at a tertiary oncology center between 2021 and 2026. Patients presenting with a suspicious ovarian mass (Group A) or requiring restaging after adnexectomy for confirmed EOC (Group B) underwent SLN mapping using a hybrid ^99m^Tc–ICG tracer injected into the infundibulopelvic (IPL) and/or utero-ovarian ligament (UOL). SLNs were identified using gamma detection and near-infrared fluorescence imaging. All malignant cases underwent complete surgical staging including systematic pelvic and para-aortic lymphadenectomy. SLNs were ultrastaged and compared with the final nodal status. **Results**: Forty patients were included; 20 (50%) had malignant tumors. The overall SLN detection rate was 92.5% (37/40), with 100% in malignant cases. Among malignant tumors, 3/20 (15%) had metastatic SLNs, all accurately detected (false-negative rate 0%). Sensitivity and negative predictive value were 100%. Combined pelvic and para-aortic drainage was the most frequent pattern (75%). **Conclusions**: SLN mapping may represent a feasible and potentially accurate staging strategy in apparent early-stage EOC. In the present study, a hybrid ^99m^Tc–ICG tracer was associated with high detection rates and complete concordance with final nodal status. These findings support further multicenter validation to define its potential role as an alternative to systematic lymphadenectomy.

## Simple Summary

Accurate evaluation of lymph node status is essential in early-stage epithelial ovarian cancer because it helps determine whether the disease has spread and guides further treatment. However, the current standard approach—systematic lymphadenectomy—can cause complications without clearly improving survival. This study explores a less invasive alternative called sentinel lymph node mapping using a new hybrid tracer that combines two detection methods into a single substance to improve accuracy during surgery. The aim was to assess whether this technique can reliably identify the first lymph nodes most likely to contain cancer cells. The results showed high detection rates and excellent agreement with final pathology findings, suggesting this method may help reduce the need for extensive lymph node removal. If confirmed in larger studies, this approach could improve surgical staging while reducing complications for patients with early-stage ovarian cancer.

## 1. Introduction

Epithelial ovarian cancer (EOC) is the third most common gynecological malignancy and remains the leading cause of mortality among these cancers, largely due to its often-asymptomatic course and the high proportion of patients diagnosed at an advanced stage, which is associated with poor prognosis [1,2].

Although early-stage disease (FIGO I–II), confined to the ovaries, fallopian tubes, or pelvis, accounts for approximately 20–25% of all cases [2,3], accurate staging is essential because up to one quarter of patients ultimately undergo upstaging due to occult extra-ovarian disease [4].

Lymph node (LN) assessment plays a pivotal prognostic role, as nodal metastases are identified in 10–20% of apparently early-stage EOC cases and directly influence decisions regarding adjuvant chemotherapy and/or maintenance therapy [4,5].

Current clinical guidelines recommend systematic pelvic and para-aortic lymphadenectomy for nodal assessment [1,6]. However, this procedure is associated with significant intra- and postoperative morbidity—including lymphocele, vascular or nerve injury, chylous ascites, and chronic lymphedema—without demonstrating a survival benefit in randomized clinical trials [5,7].

The sentinel lymph node (SLN) concept is based on the identification of the first lymphatic station to receive drainage from a primary tumor. This LN is considered the most likely initial site of metastatic spread, and its pathological assessment serves as a surrogate for the status of the entire regional nodal basin. Consequently, the absence of metastatic involvement in the SLN reliably predicts the likelihood that the remaining lymph nodes within the same anatomical territory are free of disease.

SLN biopsy has demonstrated high diagnostic accuracy in gynecologic malignancies across several prospective trials including FIRES, SENTICOL, SENTIX, SHREC and GROINSS studies [8,9,10,11,12]. Translating this approach to EOC is conceptually appealing; however, its use remains investigational due to the complexity of ovarian lymphatic drainage and the methodological heterogeneity observed across published studies [13].

Recent studies [14,15,16,17] have evaluated the feasibility of SLN mapping in early-stage EOC, using different tracers and injection techniques to improve detection accuracy. Despite these encouraging findings, the current evidence is derived from small series and early-phase studies, leaving key questions unresolved—including the optimal injection strategy and the most effective tracer modality.

In this context, a hybrid tracer, such as the combined technetium-99m nanocolloid and indocyanine green (^99m^Tc–ICG), used in the present study, could offer a potentially advantageous balance between real-time fluorescence visualization and stable radiotracer localization. This dual-modality approach may help improve SLN detection rates and reliability in apparent early-stage EOC. Unlike previously published dual-tracer approaches based on sequential tracer administration, the present study evaluates a true hybrid molecular tracer that combines a radiocolloid and fluorescence within a single compound.

Therefore, this study aimed to evaluate the feasibility, diagnostic accuracy, and procedural safety of SLN mapping using a hybrid ^99m^Tc–ICG tracer in patients with apparent early-stage EOC.

## 2. Materials and Methods

### 2.1. Study Group

A prospective, observational, descriptive, single-center study was conducted at Son Espases University Hospital between January 2021 and January 2026. Patients with clinically suspected early-stage EOC were eligible, including two subgroups: Group A, patients presenting with a suspicious ovarian mass without prior histological diagnosis, and Group B, patients with histologically confirmed malignant EOC determined by frozen-section after adnexectomy, all of whom subsequently underwent surgical staging. Physical gynecological examination by an expert oncologic gynecologist and thorax-abdomen-pelvic computed tomography (TAP-CT) were performed on all patients before surgery.

Inclusion and exclusion criteria are summarized in Table 1. All subjects provided their documented informed consent for inclusion before participating in this study. The study was conducted in accordance with institutional ethical standards and the principles of the Declaration of Helsinki, and the protocol was approved by the Ethics Committee of the Balearic Islands (IB 4800/22 PI).

### 2.2. Study Endpoints

The primary endpoint was the SLN detection rate and diagnostic accuracy using the hybrid ^99m^Tc–ICG tracer in patients with a suspicious or histologically confirmed EOC, using the final nodal status after systematic pelvic and para-aortic lymphadenectomy as the reference standard. Secondary endpoints included a detailed characterization of lymphatic drainage pathways, with assessment of the anatomical distribution of SLNs within the pelvic and para-aortic nodal basins; the number of SLNs per patient by anatomical region; the diagnostic performance of SLN mapping, determined by sensitivity and negative predictive value (NPV); and the comparison of the SLN detection rate reported in the literature.

### 2.3. Injection Site and Sentinel Lymph Node Mapping Technique

Tracer injection was performed either before an adnexectomy or after prior adnexectomy, depending on whether the patient corresponded to Group A (pre-adnexectomy) or Group B (post-adnexectomy) as defined below.

Tracer injection was performed according to previous gynecological surgery (hysterectomy and/or adnexectomy) and tumor laterality. In unilateral tumors, injections were performed on the affected side, whereas in bilateral tumors, injections were performed bilaterally. In patients without prior hysterectomy or adnexectomy, the tracer was injected into the infundibulopelvic ligament (IPL) and the utero-ovarian ligament (UOL). In patients with prior hysterectomy, injections were limited to the IPL stump(s). In patients with prior adnexectomy, injections were performed at the IPL and at the residual UOL stump (approximately 1–2 cm from the coagulated margin). In patients with both prior hysterectomy and adnexectomy, injections were limited to the available IPL stump(s).

SLN detection was performed using a hybrid tracer composed of 2 mL of diluted ICG (ICG; 2.5 mg/mL) added to the technetium vial of ^99m^Tc-nanocolloid. The tracer was injected subperitoneally (keeping the needle superficial to minimize the risk of vascular spread) at two anatomical sites: the proximal IPL and the UOL (if the uterus is in situ). A total of 37–55 MBq in 0.2 mL of ^99m^Tc-nanocolloid was injected at each site using a 22-gauge needle. Following injection, a waiting period of approximately 20–30 min was allowed to facilitate lymphatic migration.

Lymphatic drainage pathways were systematically explored in the pelvic and para-aortic regions. SLN detection was performed using a combined approach integrating gamma-probe guidance (Dilon Navigator, Dilon Technologies Inc., Newport News, VA, USA) to detect the radioactive signal of ^99m^Tc and near-infrared fluorescence imaging to visualize ICG-positive lymph nodes during laparoscopic (Karl Storz Endoscope, Tuttlingen, Germany), robotic (da Vinci^®^ Surgical System Xi, Intuitive Surgical Inc., Sunnyvale, CA, USA), or open surgery (Stryker Corporation, Kalamazoo, MI, USA).

Both modalities were used complementarily throughout the procedure to assess the same lymphatic drainage pathway. SLNs were defined as LNs showing both fluorescence signal and increased radioactive uptake detected by the gamma probe, and only nodes fulfilling both criteria were considered true SLNs.

Overall mapping feasibility was calculated using the entire study cohort as the denominator. Drainage patterns and anatomical distribution of SLNs were analyzed only among patients with successful lymphatic mapping. Basin-based detection rates were calculated according to the number of anatomical basins at risk based on tumor laterality.

### 2.4. Surgical Procedure

Two clinical scenarios were considered in this study. First, patients underwent exploratory surgery through either a minimally invasive approach (laparoscopic or robotic) or laparotomy. The surgical approach was individualized following multidisciplinary discussion, considering tumor size, imaging characteristics, previous surgical history, anticipated risk of tumor rupture, surgeon experience, and overall oncologic feasibility.

If no macroscopic extra-pelvic dissemination was observed, tracer injection was performed:

Group A: patients presenting with a suspicious adnexal mass, tracer injection was performed with the ovarian mass in situ. Adnexectomy was performed, followed by intraoperative frozen section evaluation to determine histology. SLN excision is only performed after histological confirmation of invasive EOC. In contrast, if the intraoperative result is benign or borderline (BL), lymphatic drainage pathways and the potential SLN will be explored and identified, but no excision will be undertaken.

Group B: patients with prior adnexectomy and confirmed EOC. Tracer injection was performed as described in Section 2.3.

In both cohorts, in invasive malignancy, full surgical staging—including hysterectomy, contralateral adnexectomy, omentectomy, bilateral pelvic and para-aortic lymphadenectomy, and appendectomy in mucinous carcinomas—was completed following current guidelines [6]. In BL tumors, staging was performed without systematic lymphadenectomy, as routine nodal dissection is not recommended.

The patients were followed for a 30-day period, during which all surgical events were documented, including intraoperative and postoperative complications, and any tracer-related adverse effects occurring after the procedure [18].

### 2.5. Pathological Assessment

Excised SLNs were pathologically evaluated following an ultrastaging protocol (all SLNs were formalin-fixed, paraffin-embedded, and examined through serial sectioning with Hematoxylin–Eosin staining and mixed-cytokeratin immunohistochemistry to detect micrometastases or isolated tumor cells). LNs retrieved during systematic lymphadenectomy were evaluated using standard pathological protocols with routine H&E staining.

SLN tumor burden was classified according to a modified version of the American Joint Committee on Cancer (AJCC, 9th edition) criteria applied to axillary lymph nodes in breast cancer, as commonly adopted in sentinel lymph node ultrastaging protocols in gynecologic oncology [19].

### 2.6. Data Collection

Data collection was carried out through the review of the electronic medical records available in the clinical information system of Son Espases University Hospital (Millennium, Cerner Corporation, Kansas City, MO, USA). Sociodemographic, pre-, intra-, and postoperative variables were obtained for all women who fulfilled the inclusion criteria. All extracted information was encrypted and integrated into a password-protected database created specifically for this study.

### 2.7. Statistical Analysis

A descriptive analysis of all variables was performed to characterize the study population, using frequencies and percentages for qualitative variables and measures of central position and dispersion for quantitative variables. Quantitative variables with a normal distribution were expressed as mean and standard deviation (SD), whereas those with a non-normal distribution were reported as median and interquartile range (IQR). Categorical variables were presented as absolute numbers and percentages. Regarding group comparisons, t tests or ANOVA were used for approximately normally distributed continuous variables; the Mann–Whitney test and the Kruskal–Wallis test were applied for non-normally distributed continuous variables; and chi-squared or Fisher’s exact tests were used for categorical variables.

This was an exploratory pilot feasibility study designed to evaluate the feasibility and detection rate of sentinel lymph node mapping in early-stage EOC. No formal sample size calculation was performed. All consecutive eligible patients treated during the study period were included in the analysis.

Diagnostic performance metrics included sensitivity, negative predictive value (NPV), and false-negative (FN) rate, calculated using final nodal status after systematic lymphadenectomy as the reference standard. A value of *p* < 0.05 was considered statistically significant. Statistical analyses were conducted using R, version 4.5.2.

Statistical analysis was performed at the Health Research Institute of the Balearic Islands (IdISBa).

## 3. Results

### 3.1. Patients’ Characteristics

A total of 40 patients were included between January 2021 and 2026. Patient enrollment showed a relatively even distribution across the study period, peaking in 2023 (25%) and 2024 (22.5%), with no marked fluctuations in the remaining years. Baseline clinical and surgical characteristics of the study population are summarized in Table 2.

**Figure 1:**
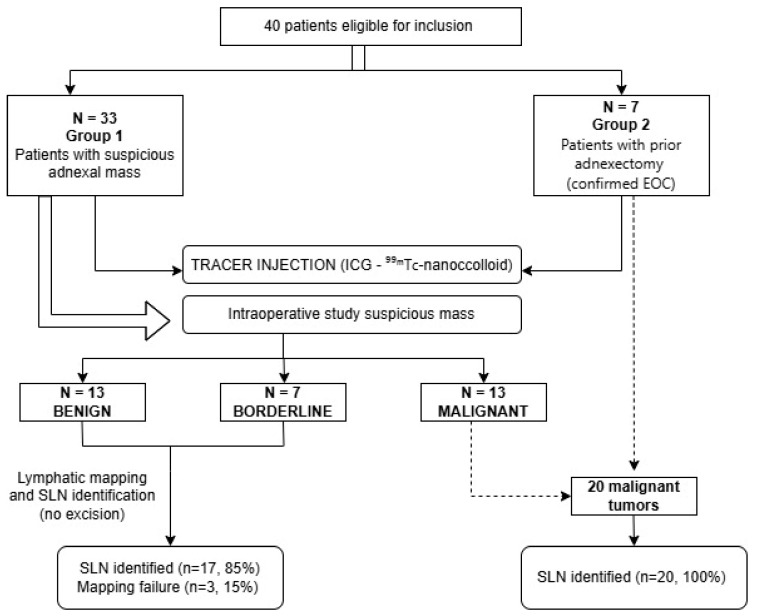
Study flow diagram of patient inclusion.

The distribution of the included patients by the study group is shown in the Study Flow Diagram (Figure 1): 33 (82.5%) in the primary surgery group and 7 (17.5%) in the re-staging surgery group. Final pathology showed 20/40 (50.0%) malignant, 13/40 (32.5%) benign, and 7/40 (17.5%) BL tumors.

### 3.2. Sentinel Lymph Node Detection and Mapping Distribution

Of the 40 patients included, tracer injection was performed with the ovarian mass in situ in 33 cases (82.5%), whereas in the remaining 7 patients (17.5%), injection was performed into the IPL and/or UOL stumps following prior adnexectomy during delayed re-staging surgery.

According to the study protocol, only LN showing both fluorescence signal and radioactive uptake were classified as SLN. All sentinel lymph nodes included in the analysis fulfilled these criteria.

Lymphatic drainage and SLN detection were assessed in all cases. Overall, at least one SLN was successfully identified in 37 patients, yielding an overall detection rate of 92.5%. SLN was not identified in three cases (7.5%), corresponding to one BL ovarian tumor and two benign tumors. In two of these failure procedures, lymphatic drainage was not visualized, whereas in one case (2.5%), intraoperative tracer spillage into the peritoneal cavity was observed.

Combined pelvic and para-aortic drainage was observed in 28/37 (75.7%), whereas isolated pelvic and isolated para-aortic drainage occurred in 5/37 (13.5%) and 4/37 (10.8%), respectively. All SLNs demonstrated drainage to the ipsilateral side of the tumor. Pelvic and para-aortic SLN were identified in 89.2% and 86.5% of patients with successful mapping, respectively. When calculated per anatomical basin at risk according to tumor laterality, the pelvic basin-based detection rate was 87.8%, whereas the para-aortic basin-based detection rate was 80.0%.

The total number of SLNs removed was 80, with a median per patient of 2 (IQR 2–3) in the right pelvis, 1 (IQR 1–2) in the left pelvis, and 1 (IQR 1–2) in the para-aortic region.

Exploratory comparisons did not identify statistically significant differences in SLN drainage patterns before versus after adnexectomy (pelvic only: 5/33 [16.7%] vs. 0/7 [0%]; combined pelvic and para-aortic: 23/33 [76.7%] vs. 5/7 [71.4%]; isolated para-aortic: 2/33 [6.7%] vs. 2/7 [28.6%]; *p* = 0.13).

**Figure 2:**
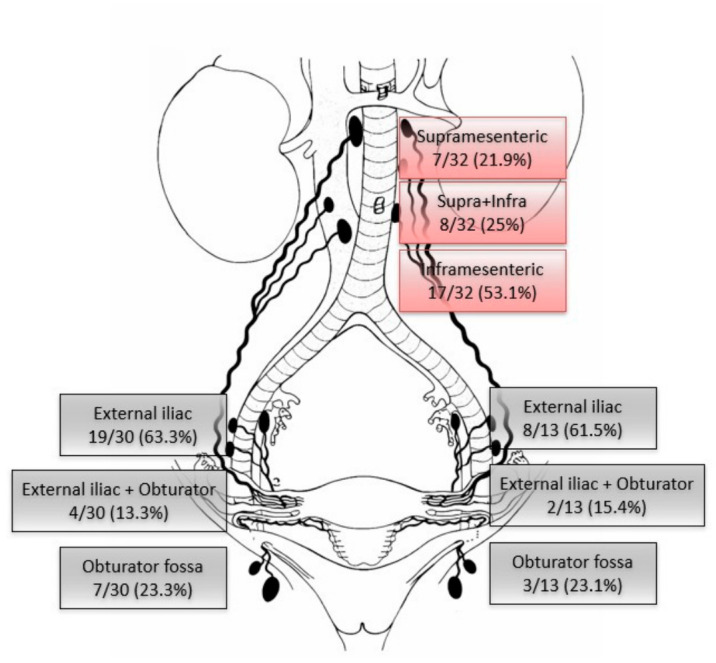
SLN anatomical distribution.

All identified SLNs were classified according to their anatomical location. Pelvic SLNs were predominantly located along the external iliac vessels, followed by the obturator fossa. Para-aortic SLNs were predominantly inframesenteric (Figure 2).

SLN removal was performed only in patients with confirmed malignancy (22/40; 55%); however, malignancy was confirmed in 20 cases on final histopathology. Two cases initially considered suspicious for invasive disease on intraoperative assessment were ultimately diagnosed as borderline tumors and excluded from the final malignant cohort. Among these, the median number of retrieved SLNs was 3 (IQR 2–5). In cases with an intraoperative diagnosis of BL or benign (18/40; 45%), lymphatic migration was assessed without excision, using a gamma probe and a mobile ICG gamma camera.

### 3.3. Subgroup of Malignant Tumors

Among the 20/40 (50%) patients with malignant tumors, prior adnexectomy and hysterectomy were present in 7/20 (35.0%) and 2/20 (10.0%), respectively. In this subgroup, baseline demographic and disease-related characteristics are presented in Table 3. Detailed anonymized patient-level clinicopathological and surgical data for all malignant cases are provided in Supplementary Tables S1 and S2.

After intraoperative assessment and SLN mapping, complete surgical staging was performed, comprising hysterectomy, contralateral salpingo-oophorectomy, pelvic and para-aortic lymphadenectomy, omentectomy, and peritoneal cytology.

Within the 20 malignant cases, SLN detection was achieved in all cases (100%). In the malignant subgroup, pelvic and para-aortic detection rates reached 90% and 85%, respectively; however, patient-based detection rates in the overall cohort were 82.5% and 80%. Combined pelvic and para-aortic drainage occurred in 15/20 (75.0%), with isolated pelvic and isolated para-aortic drainage in 3/20 (15.0%) and 2/20 (10.0%), respectively. SLN topography mirrored the overall cohort (pelvic nodes mainly external iliac; para-aortic nodes inframesenteric). The median number of SLNs per patient was 2 (IQR, 2–3) on the right pelvis, 1 (IQR, 1–2.25) on the left pelvis, and 1.5 (IQR, 1–3) in the para-aortic region.

Overall, 17/20 (85.0%) patients had negative SLNs, and 3/20 (15.0%) had metastatic SLN involvement. All these metastases were accurately detected by the SLN technique. Metastatic SLNs were located in the right pelvis in 1/3 (33.3%) and in the para-aortic region in 2/3 (66.7%); all were macrometastases. No SLN-negative cases harbored additional nodal metastases at completion of lymphadenectomy. SLN status matched the final nodal status in all 20 malignant cases (false-negative (FN) rate: 0%), yielding a sensitivity of 100% (95% CI, 29.2–100%) and a negative predictive value of 100% (95% CI, 80.5–100%), although this estimate should be interpreted cautiously given the limited number of SLN-positive cases. No empty lymph node packets were identified.

In summary, 15% of patients with apparently early-stage disease were upstaged to advanced-stage disease (FIGO IIIA1) based on SLN metastases detected by SLN mapping. In addition, one patient (5%) was classified as FIGO stage IIIA2 due to the presence of a microscopic peritoneal implant identified during surgical staging, in the absence of nodal involvement. The three SLN-positive cases included two high-grade serous carcinomas and one clear-cell carcinoma.

No intraoperative or postoperative complications related to the tracer injection or SLN technique were observed. Regarding surgical staging, four grade II complications occurred, including two cases of postoperative fever that resolved with antibiotic therapy (one associated with a small pelvic abscess), two cases of lymphorrhea managed conservatively with dietary measures and octreotide, and one case of moderate anemia requiring intravenous iron supplementation. Additionally, one patient developed postoperative bowel obstruction due to an incarcerated trocar-site hernia after robotic surgery, requiring surgical reintervention without bowel resection (grade IIIb). A 10 mm access port was routinely used for insufflation (AirSeal^®^ system) and specimen retrieval in all robotic procedures, independently of radioguided detection, which should be considered when interpreting procedure-related complications. Notably, only one port-site hernia was observed over six years of robotic surgery at our center. No grade IV–V complications were observed.

The two cases of lymphorrhea, classified as Clavien–Dindo grade II complications, were considered directly related to lymphadenectomy and were successfully managed with conservative treatment. Both patients had malignant tumors confirmed on intraoperative assessment and therefore underwent complete pelvic and para-aortic lymphadenectomies. Only one of these two patients was ultimately found to have nodal metastasis.

### 3.4. Comparison According to Previous Adnexectomy

Comparisons between patients with (n = 7) and without (n = 33) prior adnexectomy showed no significant differences in age, tumor markers, SLN detection rates, or drainage patterns. Surgical approach differed significantly (*p* = 0.001): all patients with prior adnexectomy underwent robotic surgery, while 27/33 (81.8%) without adnexectomy required laparotomy. Tumors were significantly larger in patients without prior adnexectomy for right-sided lesions (mean, 15.4 cm vs. 7.45 cm; *p* = 0.003) and left-sided lesions (median, 6.0 cm vs. 3.0 cm; *p* = 0.036).

## 4. Discussion

This prospective study provides additional evidence supporting the feasibility and diagnostic performance of SLN mapping in apparent early-stage EOC using a hybrid tracer that combines technetium-99m nanocolloid and ICG (^99m^Tc–ICG) within a single molecular complex. To our knowledge, this is the first prospective study to evaluate a true molecular hybrid tracer as the primary mapping strategy in apparent early-stage EOC. Distinguishing it from previously published studies such as SELLY and Ucella et al. [16,17], which evaluated ICG as a single tracer, and from both MELISA and SENTOV I [14,20], where dual tracers were administered separately rather than as a hybrid compound.

Hybrid tracers may improve nodal retention and reduce secondary migration compared with sequential dual-tracer protocols, potentially enhancing the identification of primary lymphatic pathways. Consistent with this hypothesis, the proportion of simultaneous pelvic and para-aortic drainage observed in our cohort (75.7%) appears higher than that reported in previous prospective series, where dual-territory mapping ranged between approximately 19% and 44%, suggesting improved simultaneous mapping of both lymphatic pathways with the hybrid tracer approach.

In our cohort, SLN detection was achieved in 92.5% of all patients and in 100% of those with confirmed malignancy. Among malignant cases, SLN status showed complete concordance with final nodal assessment (FN = 0%), resulting in a false-negative (FN) rate of 0% and yielding both sensitivity and NPV of 100%. Although these estimates should be interpreted cautiously because of the limited number of malignant tumors and SLN-positive cases (3/20), they are consistent with results reported in recent meta-analyses demonstrating NPVs approaching 100% and sensitivities above 90% when dual-tracer strategies combined with ultrastaging protocols are used [15].

From a clinical perspective, the usefulness of an SLN mapping technique depends primarily on two key parameters: detection rate (DR) and diagnostic accuracy (DA). A high DR minimizes the proportion of patients requiring systematic lymphadenectomy because of mapping failure, whereas a high DA ensures that the SLN reliably reflects the true nodal status. Both parameters are essential to determine whether SLN mapping can safely replace systematic lymphadenectomy in early-stage EOC staging algorithms.

Among malignant cases, SLN mapping showed high region-specific detection rates, with pelvic SLN detection achieved in 90% of patients and para-aortic SLN detection in 85%. These findings suggest that SLN mapping could potentially avoid systematic lymphadenectomy in the majority of patients, with only approximately 10–15% requiring completion lymphadenectomy, pending confirmation in larger prospective validation cohorts.

All patients with nodal metastases were correctly identified by the SLN technique, whereas no additional metastatic nodes were found in non-SLN specimens. Therefore, if validated in larger prospective studies, SLN mapping could potentially spare approximately 85% of patients from systematic lymphadenectomy (de-escalation surgery) while still accurately identifying those requiring nodal upstaging and adjuvant treatment. The limited number of malignant cases precludes definitive conclusions, and further validation in larger prospective studies is required.

Importantly, only two postoperative complications were directly attributable to lymphadenectomy itself, whereas the remaining adverse events were related to the overall surgical procedure. Nevertheless, these findings highlight the non-negligible morbidity associated with comprehensive surgical staging and further support the rationale for investigating SLN mapping as a strategy to reduce the morbidity of systematic lymphadenectomy while preserving accurate nodal assessment.

Although many high-grade tumors may ultimately receive adjuvant chemotherapy irrespective of nodal status, accurate LN assessment remains relevant for prognostic stratification and current FIGO staging algorithms. In this setting, SLN mapping may be a strategy to reduce the morbidity of systematic lymphadenectomy while preserving staging accuracy.

The observed differences in detection rates between pelvic and para-aortic territories highlight the importance of evaluating SLN mapping performance in a region-specific manner, particularly in malignancies characterized by complex and multidirectional lymphatic drainage such as other gynaecologic malignancies [21]. While most studies report lower pelvic SLN DR compared with para-aortic mapping [14,15,16], Lago et al. [22] and our study observed comparatively high pelvic basin-based detection rates (93% and 87.8%, respectively). The reasons underlying this discrepancy remain uncertain but may be related to differences in injection depth, tracer diffusion, or surgical standardization [17].

An interesting observation was that no cases of isolated pelvic drainage were identified among patients who had undergone prior adnexectomy. Although this subgroup was small (n = 7), most patients demonstrated combined pelvic and para-aortic drainage, while the remaining cases showed isolated para-aortic drainage. These findings may suggest that para-aortic lymphatic pathways remain preserved following adnexectomy; however, given the limited sample size, this observation should be considered hypothesis-generating and requires confirmation in larger studies.

Overall, these regional differences reflect the inherent variability of lymphatic drainage in EOC. Para-aortic mapping tends to demonstrate more consistent detection patterns across studies, whereas the pelvic basin remains technically more challenging. However, emerging evidence suggests that optimized injection strategies may theoretically reduce this gap. Notably, studies evaluating dual-tracer and dual-site injection approaches have shown concordance between cervical and UOL injections in identifying the same pelvic SLNs, indicating that cervical injection, a standardized and reproducible technique, may represent a reliable alternative access route to pelvic SLN pathways [23] and deserves further investigation.

Compared with previously published studies, our overall DR is consistent with dual-tracer approaches reported in the MELISA and slightly lower than the SENTOV trials [14,22] and appears superior to single-tracer strategies such as the SELLY trial, Ucella et al., and Lago et al. [16,17,24]. Unlike MELISA and SENTOV, which used separately administered tracers, the present study evaluated a true molecular hybrid ^99m^Tc–ICG tracer, providing prospective evidence on the feasibility of this approach in apparent early-stage EOC. These comparisons may suggest that combined radiotracer and fluorescence guidance may facilitate mapping in complex lymphatic territories such as the para-aortic region. However, because the present study did not compare individual tracers, no conclusions can be drawn regarding the superiority of the hybrid approach over ICG or ^99m^Tc alone. Sentinel lymph nodes were successfully identified using both fluorescence and radioguided detection modalities throughout the study.

A comparison of SLN detection rates and anatomical drainage patterns across prospective SLN mapping studies in early-stage epithelial ovarian cancer, including the present cohort, is summarized in Table 4.

A distinctive feature of our series is the use of a true hybrid ^99m^Tc–ICG tracer combined with low-volume ICG administration, in contrast to the 2 mL bolus typically used in SELLY-based protocols. This technical refinement may reduce excessive tracer diffusion and nonspecific retroperitoneal fluorescence, thereby reducing the likelihood of ENP retrieval and improving concordance between fluorescence-guided and radiotracer-guided detection modalities. In both studies using ICG as a single tracer, the incidence of ENP was notably elevated: 27% in the pelvic area and 7% in the para-aortic region in one series [24], and 12.5% in the multicenter cohort [16]. These findings suggest that single-tracer ICG protocols may inherently increase the risk of ENP, regardless of the surgical setting.

Our high overall SLN detection rates likely reflect a combination of factors, including the use of a hybrid tracer, a standardized surgical technique, multidisciplinary collaboration, and the previous experience of our group with SLN mapping in other gynecologic malignancies, particularly endometrial and cervical cancers, which may have contributed to optimizing the surgical technique and detection performance [26,27]. The relative contribution of each factor cannot be determined from the present study. Hybrid tracers combine real-time fluorescence imaging with the deeper tissue penetration and nodal retention of radiocolloids, potentially overcoming limitations associated with fluorescence-only mapping such as rapid diffusion and signal spillage.

Although modality-specific detection rates were not prospectively recorded, no clinically relevant discrepancies between fluorescence and radioguided detection were observed during SLN identification. Therefore, although the present study does not allow an independent assessment of the contribution of each modality, the combined approach proved feasible in routine clinical practice and was not associated with any tracer-related safety concerns.

The lymphatic drainage patterns observed in our study further reinforce the dual-pathway theory of ovarian lymphatic spread. Combined pelvic and para-aortic drainage was the most frequent pattern, emphasizing the importance of systematic evaluation of both nodal regions during staging procedures. In our series, the anatomical distribution of SLNs was consistent with known pathways, predominantly along the external iliac vessels and in the inframesenteric para-aortic region.

We did not observe significant differences in SLN detection according to the timing of tracer injection (pre- versus post-adnexectomy), although this finding should be interpreted cautiously given the limited sample size. These results suggest that both strategies are feasible within standardized SLN mapping protocols, but adequately powered prospective studies are needed to determine whether one approach provides superior reproducibility.

Comparisons between patients with and without previous adnexectomy showed no significant differences in age, tumor marker levels, SLN detection rates, or lymphatic drainage patterns, suggesting that prior adnexal removal alone may not substantially compromise lymphatic mapping reliability. These findings contrast with previous reports suggesting reduced detection performance in re-staging procedures, possibly reflecting surgically induced disruption of lymphatic pathways after prior adnexectomy [17,28,29].

In our clinical practice, large adnexal masses often necessitate laparotomy, which requires real-time intraoperative decision-making to avoid subjecting patients to two separate surgical procedures. For this reason, in our institution protocol, the tracer is typically administered before adnexectomy, aiming to minimize manipulation of pelvic structures that could potentially disrupt lymphatic channels and alter subsequent tracer migration. The theoretical advantage of this approach lies in preserving the integrity of lymphatic drainage from an intact ovary, thereby facilitating more reliable SLN visualization. Conversely, post-adnexectomy injection has the benefit of restricting tracer administration to cases where malignancy has been confirmed, potentially reducing unnecessary exposure in benign scenarios.

Regarding oncologic outcomes, metastatic SLNs were identified in 15% of malignant cases, all corresponding to macrometastases. The absence of low-volume metastases (LVM) in our cohort is most likely attributable to the limited sample size rather than to true biological differences, particularly considering previous prospective studies demonstrating clinically relevant rates of LVM detected through ultrastaging [30].

This study has some limitations. First, it represents a single-center experience with a relatively limited number of malignant cases, inherently limiting statistical power. Additionally, the inclusion of benign and borderline tumors reflects real-world clinical practice but may complicate direct comparisons with malignant-only series. Finally, a subset of patients was ultimately diagnosed with invasive EOC. Although lymphatic drainage pathways and potential SLN could be identified regardless of final histology, the clinical applicability of SLN assessment can only be evaluated in patients with invasive disease. Therefore, the oncologic implications of our findings are based on a limited number of malignant cases.

Nevertheless, several important strengths should be highlighted. This study represents one of the largest prospective single-center cohorts. The prospective design, the inclusion of 40 consecutively enrolled patients, and the use of a standardized hybrid tracer injection protocol contributed to a high overall detection rate and reproducible mapping performance. These features support the internal validity of the study and reinforce the feasibility of the hybrid tracer approach in clinical practice.

## 5. Conclusions

In conclusion, SLN mapping may represent a feasible and potentially accurate staging strategy in apparent early-stage EOC. These findings support further evaluation within multicenter prospective validation studies aimed at defining its role as a potential alternative to systematic lymphadenectomy in surgical staging algorithms for apparent early-stage EOC.

In this prospective study, a hybrid ^99m^Tc-ICG tracer achieved high detection rates and complete concordance with final nodal status. Further comparative studies are needed to determine the relative contribution of individual tracers and to clarify whether hybrid mapping approaches provide additional benefits over single-tracer techniques.

The high proportion of simultaneous pelvic and para-aortic mapping observed in this series supports the hypothesis that hybrid tracers may improve the reproducibility of dual-pathway ovarian lymphatic mapping, representing a relevant step toward standardization of SLN techniques in early-stage ovarian cancer.

## Figures and Tables

**Table 1:** Selection and exclusion criteria.

SELECTION CRITERIA
(1) Women aged ≥ 18 years
(2) Suspected or histologically confirmed epithelial ovarian carcinoma
(3) Apparently early-stage epithelial ovarian cancer (FIGO 2014 I–II)
(4) Scheduled to undergo surgical staging
(5) Provided written informed consent
**EXCLUSION CRITERIA**
(1) Evidence of extrapelvic disease at surgical exploration (FIGO stage III–IV)
(2) Body mass index > 42 kg/m^2^
(3) Known allergy to tracer components or hyperthyroidism
(4) Prior pelvic/para-aortic lymphadenectomy
(5) Prior pelvic/abdominal radiotherapy
(6) Contraindication to surgery (severe comorbidities)
(7) Failure to provide informed consent or withdrawal of consent

FIGO = International Federation of Gynecology and Obstetrics.

**Table 2:** Baseline clinical and surgical characteristics of the study population.

Variable	All Cases (N = 40)
**Mean age, years (SD)**	54.6 (9.18)
**Adnexal mass location**	
Right	24 (60%)
Left	7 (17.5%)
Bilaterally	9 (22.5%)
**Tumor size** (cm)	
Right-sided masses, mean (SD)	13.9 (6.98)
Left-sided masses, median (IQR)	6.0 (3.0–18.0)
**Serum tumor marker elevated**	
**n (%; median, range)**	
CA-125	26 (65%; 88.2 U/mL, IQR 19.2–235.0)
CA-19-9	15 (37.5%; 15.5 U/mL, IQR 4.28–99.0)
HE4	8 (20.0%; 63.0 U/mL, IQR, 44.0–79.0)
**Surgical approach** (n, %)	
Laparotomy	27 (67.5%)
Robotic	11 (27.5%)
Laparoscopic	2 (5.0%)
**Type of surgery**	
Immediate staging	33 (82.5%)
Delayed staging	7 (17.5%)
**Tracer injection site**	
Dual injection (IPL and UOL)	28 (70%)
Bilateral dual injection	9 (22.5%)
Single IPL injection (prior hysterectomy)	3 (7.5%)

SD = Standard Deviation; IQR = Interquartile Range; IPL = Infundibulo-pelvic ligament; UOL = utero-ovarian ligament.

**Table 3:** Demographic, clinical, and surgical characteristics of Malignant epithelial ovarian tumors.

Variable	Value (N = 20)
**Mean age, years (SD)**	54.4 (8.77)
**Genetic test**	
BRCA ½	3 (15%)
HRD	1 (5%)
Not mutated	7 (35%)
Not informative	2 (10%)
Not indicated	1 (5%)
Not performed	6 (30%)
**Serum tumor marker elevated**	
n (%; median, range)	
CA-125	16 (80.0%; 143 U/mL, IQR, 76.5–281.0)
CA-19-9	9 (45.0%; 19.1 U/mL, IQR, 10.2–218.0)
HE4	4 (20.0%; mean, 71.9 U/mL, SD 20.4)
**Tumor size**	
(cm, median IQR)	
Right-sided mass	11.0 (IQR 9.0–15.0)
Left-sided mass	6.06 (IQR 3.00–12.2)
**Surgical approach**	
Laparotomy	12 (60%)
Robotic	7 (35%)
Laparoscopic	1 (5%)
**Histotype, n (%)**	
Serous	7 (35%)
Endometrioid	4 (20%)
Clear cell	3 (15%)
Mix (endometrioid + clear cell)	2 (10%)
Mucinous	4 (20%)
**Tumor grade, n (%)**	
Low-grade (G1-G2)	7 (35%)
High-Grade (G3)	13 (65%)
**Final FIGO 2014 stage, n (%)**	
IA	7 (35%)
IB	1 (5%)
IC1/IC2	7 (35%)
IIB	1 (5%)
IIIA1	3 (15%)
IIIA2 (microscopic peritoneal implant)	1 (5%)
**SLN mapping rate**	20 (100%)
SLN/patient	3.00 (IQR 2.00–5.25)
**Number removed LN**	
Right pelvic LND (mean, SD)	7.95 (3.55)
Left pelvic LND (median, IQR)	6.00 (3.75–8.00)
Para-aortic LND (mean, SD)	7.65 (3.67)
**Positive lymph nodes, n (%)**	3/20 (15%)
Pelvic SLN	1 (33.3%)
Pelvic no-SLN	0 (0%)
Para-aortic SLN	2 (66.7%)
Para-aortic no-SLN	0 (0%)

SLN = sentinel lymph node; LN = lymph node; LND = lymphadenectomy; FIGO = International Federation of Gynecology and Obstetrics.

**Table 4:** Comparison of sentinel lymph node detection rates and anatomical drainage patterns among prospective mapping studies in early-stage epithelial ovarian cancer, including the HibrOv Trial cohort.

Study (Author, Year)	N	Tracer Type	Tracer Injection Timing	Malignant Histology n (%)	Overall SLN DR (%)	Pelvic DR *	Para-Aortic DR	Pelvic Only	Para-Aortic Only	Pelvic + Para-Aortic	Sensitivity FN
**SENTOV** **(Lago et al., 2021)** [25]	**30**	Dual ^99m^Tc 0.2 mL (37 MBq) + ICG 0.5 mL (1.25 mg/mL)	Post-adnexectomy stump injection prior to staging	20 (67%)	100% (30/30)	91.3%	90%	--	--	--	100% 0%
**MELISA Trial** **(Agustí et al., 2023)** [14]	**30**	Dual ^99m^Tc 0.2 mL (37 MBq) + ICG 0.2–0.5 mL (2.25 mg/mL)	^99m^Tc: Pre-adnexectomy ICG: Post-adnexectomy Prior to surgical staging	27 (90%)	90% (27/30)	44.8%	86.7%	4%	52%	44%	100% 0%
**SELLY** **(Nero et al., 2024)** [16]	**169**	Single ICG 2 mL (1.25 mg/mL)	Post-adnexectomy	107 (63%)	58.6% (99/169)	50.9%	55%	--	--	--	73.3% 4%
**Uccella et al., 2025** [17]	**36**	Single ICG 2 mL (1.25 mg/mL)	Post-adnexectomy	31 (86%)	86% (31/36)	50%	80.6%	19.4%	25.8%	54.8%	100% 0%
**HibrOv Trial**	**40**	Hybrid 0.75–2 mL ICG + ^99m^Tc 0.2 mL (37–55 MBq)	Pre-adnexectomy and prior to surgical staging	20 (50%)	92.5% (37/40)	82.5% (33/40)	80% (32/40)	13.5%	10.8%	75.7%	100% 0%

DR = detection rate; SLN = sentinel lymph node; FN = false-negative rate; ICG = indocyanine green; ^99m^Tc = technetium-99m; UOL = utero-ovarian ligament; IPL = infundibulopelvic ligament. Pelvic DR (%) *: Pelvic and para-aortic detection rates are reported according to each study definition (per patient in SENTOV, MELISA, and HibrOv trials; per mapping area in SELLY and Uccella studies). Reported per hemipelvis in SELLY and Uccella studies.

## Data Availability

The data supporting the findings of this study are available from the corresponding author (J.A.V.) upon reasonable request. The data are not publicly available due to privacy and ethical restrictions.

## Supplementary Materials

The following supporting information can be downloaded at: https://www.mdpi.com/article/10.3390/cancers18121973/s1. Table S1: Clinicopathological characteristics of the 20 malignant cases; Table S2: Surgical and sentinel lymph node mapping outcomes of the 20 malignant cases.

## Abbreviations

The following abbreviations are used in this manuscript:

AJCC	American Joint Committee on Cancer
BL	borderline tumor
CI	confidence interval
CT	computed tomography
DA	diagnostic accuracy
DR	detection rate
EOC	epithelial ovarian cancer
ENP	empty nodal packet
FN	false negative
FIGO	International Federation of Gynecology and Obstetrics
H&E	hematoxylin–eosin
ICG	indocyanine green
IQR	interquartile range
IPL	infundibulopelvic ligament
LN	lymph node
LVM	low-volume metastasis
MIS	minimally invasive surgery
NPV	negative predictive value
SD	standard deviation
SLN	sentinel lymph node
TAP-CT	thorax–abdomen–pelvic computed tomography
UOL	utero-ovarian ligament
^99m^Tc	technetium-99m
