# Supplementary material for: Hybrid 99mTc–ICG Sentinel Lymph Node Mapping in Apparent Early-Stage Epithelial Ovarian Cancer: A First Prospective Evaluation of a True Molecular Hybrid Tracer (HibrOv Trial)
Source: Cancers (Basel). 2026 Jun 17;18(12):1973. doi: 10.3390/cancers18121973 (PMC13296627; doi:10.3390/cancers18121973)
Supplement: Supplementary file 1 [file cancers-18-01973-s001.zip › cancers-4315361-supplementary.pdf]

cancers

MDPI

**Table S1**. Clinicopathological characteristics of the 20 malignant cases.

| Case | Age | Histology | Grade | Tumor side | Right tumor size (cm) | Left tumor size (cm) | FIGO |
|---|---|---|---|---|---|---|---|
| 1 | 50 | Clear-cell | High-grade | Right | 16 | | IA |
| 2 | 40 | HGSC | High-grade | Bilateral | 6 | 6 | IIIA1(ii) |
| 3 | 61 | Mixed: clear-cell/endometrioid | High-grade | Bilateral | 9 | 7 | IB |
| 4 | 64 | HGSC | High-grade | Right | 10 | | IC1 |
| 5 | 50 | HGSC | High-grade | Bilateral | 4 | 3 | IC1 |
| 6 | 55 | Endometrioid | Low-grade | Left | | 3 | IA |
| 7 | 60 | Mucinous | High-grade | Right | 10 | | IA |
| 8 | 49 | HGSC | High-grade | Bilateral | 2 | 3 | IB |
| 9 | 66 | Endometrioid | Low-grade | Right | 11 | | IA |
| 10 | 49 | Clear-cell | High-grade | Right | 20 | | IIIA1(ii) |
| 11 | 41 | HGSC | High-grade | Right | 14 | | IIIA1(ii) |
| 12 | 45 | Endometrioid | Low-grade | Right | 10 | | IC1 |
| 13 | 49 | HGSC | High-grade | Bilateral | 16 | 6 | IIIA2 |
| 14 | 44 | Mixed: clear-cell/endometrioid | High-grade | Right | 11 | | IC1 |
| 15 | 56 | Mucinous | Low-grade | Left | | 25 | IA |
| 16 | 66 | HGSC | High-grade | Right | 9 | | IC1 |
| 17 | 51 | Mucinous | Low-grade | Right | 14 | | IA1 |
| 18 | 60 | Clear-cell | High-grade | Right | 15 | | IIB |
| 19 | 62 | Endometrioid | Low-grade | Left | | 20 | IC2 |
| 20 | 69 | Mucinous | Low-grade | Right | 35 | | IA |

**Table S2.** Surgical and sentinel lymph node mapping outcomes of the 20 malignant cases.

| Case | Surgery | Upfront vs restaging | Prior adnexectomy | Prior hysterectomy | Injection technique | SLN detected | SLN location | Total SLNs re-trieved | SLN positive | Complication |
|---|---|---|---|---|---|---|---|---|---|---|
| 1 | Laparotomy | Upfront | No | No | Double | Yes | Pelvic | 1 | No | No |
| 2 | Laparoscopy | Upfront | No | No | Double bilateral | Yes | Pelvic+Paraaortic | 6 | Yes (Paraaortic) | No |
| 3 | Laparotomy | Upfront | No | No | Double bilateral | Yes | Pelvic | 2 | No | No |
| 4 | Robotic | Restaging | Yes | No | Double | Yes | Pelvic+Paraaortic | 2 | No | No |
| 5 | Robotic | Restaging | Yes | No | Double bilateral | Yes | Pelvic+Paraaortic | 7 | No | No |
| 6 | Robotic | Restaging | Yes | Yes | Simple | Yes | Paraaortic | 2 | No | No |
| 7 | Robotic | Restaging | Yes | Yes | Simple | Yes | Paraaortic | 3 | No | No |
| 8 | Robotic | Restaging | Yes | No | Double bilateral | Yes | Pelvic+Paraaortic | 6 | No | No |
| 9 | Laparotomy | Upfront | No | No | Double | Yes | Pelvic+Paraaortic | 3 | No | No |
| 10 | Laparotomy | Upfront | No | No | Double | Yes | Pelvic+Paraaortic | 5 | Yes (Paraaortic) | No |
| 11 | Laparotomy | Upfront | No | No | Double | Yes | Pelvic | 2 | Yes (Pelvic right) | Lymphorrea |
| 12 | Laparotomy | Upfront | No | No | Double | Yes | Pelvic+Paraaortic | 5 | No | No |
| 13 | Laparotomy | Upfront | No | No | Double bilateral | Yes | Pelvic+Paraaortic | 6 | No | No |
| 14 | Robotic | Restaging | Yes | No | Double | Yes | Pelvic+Paraaortic | 2 | No | No |
| 15 | Laparotomy | Upfront | No | No | Double | Yes | Pelvic+Paraaortic | 2 | No | Lymphorrea |
| 16 | Robotic | Restaging | Yes | No | Double | Yes | Pelvic+Paraaortic | 3 | No | Port-site hernia |
| 17 | Laparotomy | Upfront | No | No | Double | Yes | Pelvic+Paraaortic | 4 | No | No |
| 18 | Laparotomy | Upfront | No | No | Double | Yes | Pelvic+Paraaortic | 3 | No | No |
| 19 | Laparotomy | Upfront | No | No | Double | Yes | Pelvic+Paraaortic | 7 | No | No |
| 20 | Laparotomy | Upfront | No | No | Double | Yes | Pelvic+Paraaortic | 2 | No | No |

Abbreviations: SLN, sentinel lymph node; FIGO, International Federation of Gynecology and Obstetrics; HGSC, high-grade serous carcinoma. Cases are numbered sequentially for anonymization purposes and do not correspond to the order of inclusion. Injection technique**:** Simple = injection in the ovarian ligament only;

https://doi.org/10.3390/cancers18121973

Double = injection in both the ovarian ligament and ipsilateral infundibulopelvic ligament; Double bilateral = bilateral injection in both ovarian and infundibulopelvic ligaments.

https://doi.org/10.3390/cancers18121973

**Disclaimer/Publisher's Note:** The statements, opinions and data contained in all publications are solely those of the individual author(s) and contributor(s) and not of MDPI and/or the editor(s). MDPI and/or the editor(s) disclaim responsibility for any injury to people or property resulting from any ideas, methods, instructions or products referred to in the content.

https://doi.org/10.3390/cancers18121973
